# Maternal mental health modifies the association of food insecurity and early child development

**DOI:** 10.1111/mcn.12997

**Published:** 2020-04-30

**Authors:** Jéssica Pedroso, Gabriela Buccini, Sonia Isoyama Venancio, Rafael Pérez‐Escamilla, Muriel Bauermann Gubert

**Affiliations:** ^1^ Postgraduate Program in Human Nutrition, Center for Epidemiological Studies in Health and Nutrition ‐ NESNUT University of Brasilia Brasilia Brazil; ^2^ Social and Behavioral Sciences, Yale School of Public Health Yale University New Haven Connecticut; ^3^ Center for Epidemiological Studies in Health and Nutrition ‐ NESNUT University of Brasilia Brasilia Federal District Brazil; ^4^ State Health Department of Sao Paulo Health Institute Sao Paulo Brazil; ^5^ Department of Nutrition, Center for Epidemiological Studies in Health and Nutrition ‐ NESNUT University of Brasilia Brasilia Brazil

**Keywords:** anxiety, child development, depression, food insecurity, maternal behaviour, mental health, mother–child relations

## Abstract

We examined the association between household food insecurity and early child development and whether or not maternal depression and anxiety modifies this association. The cross‐sectional study included 468 mother–infant pairs recruited at primary health centers of the Federal District, Brazil. Mothers answered a questionnaire that evaluated early child development (outcome), household food insecurity (independent variable), maternal depression and trait anxiety (effect modifiers). Variables were collected with validated questionnaires for the Brazilian population. Pearson's *χ* ^2^ test and logistic regression analyses were conducted. Infants who lived in a moderate or severe food insecure household had 2.52 times (95% confidence interval [CI] [1.13, 5.65]) the odds of having early child development delays compared with infants in secure households. Maternal depression and anxiety modified the strength of association between household food insecurity and early child development, which is an innovative finding. Among infants with depressed mothers, those experiencing mild (adjusted odds ratio [aOR] 3.33, 95% CI [1.17, 9.46]) and moderate/severe household food insecurity (aOR 10.13, 95% CI [2.18, 47.10]) had higher odds of having early child development delays, compared with infants in food secure households. Among infants with both anxious and depressed mothers, these associations were even stronger for mild (aOR 4.69, 95% CI [1.41, 15.59]) and moderate/severe household food insecurity (aOR 16.07, 95% CI [2.70, 95.66]). In conclusion, household food insecurity is a risk factor for early child development delays, and this association is modified by maternal depression and anxiety. Future studies should evaluate the impact of intervention packages that address maternal depression and anxiety and household food insecurity on preventing early child development delays.

**Key messages**

- Household food insecurity (HFI) is an independent risk factor for early child development (ECD) delays in low‐income settings in Brazil.
- Maternal depression increases the risk of delayed ECD associated with HFI.
- Multisectoral interventions aiming to promote ECD should address caregiver's depression, anxiety and HFI.
- Paediatricians, primary health care workers and ECD programme workers should routinely screen for HFI and caregiver's depression and anxiety.

## 1 INTRODUCTION

Poor early child development (ECD) is related to worse academic performance, poverty in adulthood and the intergenerational transmission of poverty (Grantham‐McGregor et al., 2007; Lu, Black, & Richter, 2016; Britto et al., 2017; Milner, Fiorella, Mattah, Bukusi, & Fernald, 2017). For these reasons, investing in ECD and addressing its risk factors such as household food insecurity (HFI) is key to achieving the Sustainable Development Goals (Lu et al., 2016; Pérez‐Escamilla, 2017; Pérez‐Escamilla & Vianna, 2012) and efforts should be made to monitor ECD at a population level globally (World Bank, 2017).

ECD is influenced by social, economic, cultural, biological, physiological, behavioural and psychological factors (Black et al., 2017). HFI, defined here as not having stable access to adequate quantity and quality of foods to lead a healthy productive life, is a determinant of ECD and affects almost one quarter of households in Brazil (Instituto Brasileiro de Geografia e Estatística, 2013; Pérez‐Escamilla & Vianna, 2012; Zaslow et al., 2009). HFI can negatively influence the child's psycho‐emotional, social, academic, and cognitive development through at least two pathways involving nutrition and psycho‐emotional stress (Figure S1) (King, 2018; Milner et al., 2017; Pérez‐Escamilla & Vianna, 2012).

In the first pathway, HFI impacts ECD through dietary patterns characterized by poor dietary quality and/or lack of sufficient calories. The resulting macronutrient and micronutrient deficiencies in turn can affect the development of the children's brain and consequently their developmental skills (Huang, Potochnick, & Heflin, 2018; Johnson & Markowitz, 2018a; King, 2018; Pérez‐Escamilla & Vianna, 2012). In the second pathway, caregiver's depression and anxiety resulting from HFI can lead to poor stimulation, interactions and attachment with the children, also negatively impacting the children's development (Herba, Glover, Ramchandani, & Rondon, 2016; Huang et al., 2018; Johnson & Markowitz, 2018a; King, 2018; Pérez‐Escamilla & Vianna, 2012). In addition, caregiver's poor mental health can also result on HFI, given that this relation may be bidirectional (Figure S1) (Gebreyesus, Endris, Hanlon, & Lindtjørn, 2018; Weigel, Armijos, Racines, Cevallos, & Castro, 2016).

Promoting ECD during the first years of life is especially important, as this is the period of life when humans rely almost entirely on their caregivers to meet their stimulation and feeding needs (Johnson & Markowitz, 2018a; Sigla, Kumbakumba, & Aboud, 2015). There is a scarcity of studies that have previously evaluated the relationship between HFI and ECD in low‐ and middle‐income countries (Oliveira et al., 2020), and to our knowledge, no previous study has attempted to find out if maternal depression and anxiety modifies this relationship. Thus, the present study aimed to evaluate the association between HFI and ECD and whether or not maternal depression and anxiety modifies this association in a low‐income setting in Brazil. We hypothesized a priori that both maternal anxiety and depression would modify this association.

## 2 METHODS

We conducted a cross‐sectional study with 468 mother–infant pairs at 20 urban primary health centers (PHCs) of the Federal District, Brazil. This analysis is a part of a larger observational study that evaluates the association of maternal depression and anxiety with infant's health and nutrition. The minimum sample size (*n* = 461) was calculated for this larger study based on the prevalence of maternal depression (50%) to detect an association between maternal depression, HFI and ECD, considering a confidence interval (CI) of 10%, an alpha of 5% and a loss of up to 20% due to incomplete data.

We included in the study mothers who were at least 20 years old with children who were between 6 and 12 months. In case of twins, we included the older sibling. Of those recruited in the study (*n* = 623), 79 mothers refused to participate and 45 did not complete the assessment, resulting in 499 mother–child dyads with complete data. We excluded dyads in which infants presented pathologies that could directly or indirectly affect ECD (*n* = 25) such as anaemia, asthma, bronchitis, glucose 6‐phosphate dehydrogenase (G6PD) deficiency, cardiopathy, phenylketonuria, seizures, syphilis, mononucleosis, meningitis and jaundice. Pairs with missing data on the outcome (ECD) or the independent variable (HFI) (*n* = 6) were also excluded.

Any mother–infant pairs that met the inclusion criteria were invited to participate in the study, until all the possible participants from that PHC were recruited. The recruitment was systematic according to attendance to immunization or growth and development monitoring sessions. The first author trained and supervised undergraduate nutrition students who applied the questionnaires. Only the part of the questionnaire that evaluated depression and anxiety was self‐administered. Data collection occurred from March to September of 2018 during all the collected period according to the interviewer's schedule, with recruitment taking place on days of immunizations, growth and development monitoring sessions at PHC.

Regular meetings, training, mentoring supervision and a quality control assurance process ensured that data were collected according to protocol. We performed quality control through telephone calls made to 5% of the mothers participating in the study. We asked three sociodemographic questions from each mother, and no inconsistency was found between the data obtained through the call and through questionnaire answers. All study participants were clearly explained the study and signed the study's consent form.

### 2.1 Variables

The questionnaire included ECD (outcome); HFI (independent variable); and maternal depression and anxiety (effect modifiers). Mothers also answered questions regarding the following covariates: household socio‐economic and demographic characteristics; maternal characteristics; infant's characteristics; infant feeding; bottle; and pacifier use. Variables were selected based on previous empirical evidence or conceptual considerations (Pérez‐Escamilla & Vianna, 2012). The questionnaire was pretested and verified in a pilot study with 30 mothers in a PHC not included in the study.

#### 2.1.1 Outcome

ECD was assessed using the Early Childhood for Healthy Adults (PIPAS) questionnaire that has been found to be valid and reliable for Brazilian children up to 59 months (Venancio, 2018; Venancio et al., 2019). PIPAS is a population‐level measure for assessing the overall development of children aged 0–5 years, consisting in a population assessment instrument and not a diagnosis tool for ECD delay. PIPAS was selected because in addition to having been developed and validated for the Brazilian population, it is easy to administer based on caregiver's reports (Venancio, 2018; Venancio et al., 2019; World Bank, 2017). As expected, PIPAS items vary according to infant's age: birth to 6 months includes nine items, between 7 and 9 months includes nine items and between 10 and 12 months includes 12 items (Table S1) (Venancio, 2018; Venancio et al., 2019).

In response to PIPAS questionnaire, the caregiver was asked to report if the child was able to complete specific tasks in four ECD domains (motor, language, cognition and socioemotional), and the answers were used to generate a score that reflects the overall ECD status.

Standardized percentile scores were generated from PIPAS based on the percentage of developmental milestones attained. Based on exploratory analyses of the ECD score distribution and on the risk for developmental delay found in previous studies conducted with Brazilian children, the 30th percentile of the score's distribution was selected as the cut‐off point used for identifying children screening positive for higher risk of ECD delays (Halpern, Giugliani, Victora, Barros, & Horta, 2000; Maria‐Mengel & Linhares, 2007; Biscegli, Polis, Santos, & Vicentin, 2007; Coelho, Ferreira, Sukiennik, & Halpern, 2016; Venancio, 2018; Oliveira et al., 2019; Araujo, Quadros, Murata, & Israel, 2019).

#### 2.1.2 Independent variable

HFI was measured with the Brazilian Food Insecurity Measurement Scale (EBIA). EBIA was adapted from the US Household Food Security Survey Module (HFSSM) and has been validated for the Brazilian population (Bickel, Nord, Price, Hamilton, & Cook, 2000; Pérez‐Escamilla et al., 2004; Segall‐Corrêa & Marin‐León, 2009). We used the 14‐item EBIA version previously validated in Brazil (Segall‐Corrêa, Marin‐León, Melgar‐Quinonez, & Pérez‐Escamilla, 2014) that measures, through perception and experience, the situation with regard to dietary quality and quantity for adults and children living in the household. Based on the additive score, households were classified according to the level of household food insecurity severity using the following recommended cut‐off points: food secure (0 points—access to food in adequate quantity and quality without having the access to other essential needs compromised); mildly food insecure (1 to 5 points—concern about the future availability of food at the household, affecting mainly food quality, not quantity); and moderately or severely food insecure (6 to 14 points—reduced quantity of food in the household, compromising the food intake and, in the most severe cases, leading to hunger) (Instituto Brasileiro de Geografia e Estatística, 2013). The moderate and severe categories were combined as previously recommended (Panigassi et al., 2008; Santos, Silveira, Longo‐Silva, Ramires, & de Menezes, 2018).

#### 2.1.3 Effect modifiers

Effect modification occurs when the relationship between the independent variable and the outcome is modified by a third variable, that is, the effect modifier (Jupiter, 2016). The two effect modifiers examined in this study were maternal depression and anxiety. Maternal depression was assessed using the Beck Depression Inventory II (Beck, Steer, & Brown, 1996), which has been validated in Brazil (Gomes‐Oliveira, Gorenstein, Lotufo Neto, Andrade, & Wang, 2012). It is a self‐administered questionnaire, composed of 21 items. As recommended, mothers were classified as having symptoms of depression when the score was equal or higher than 14 out of 63 points (Tuovinen et al., 2018; Wolford et al., 2017).

Anxiety was evaluated using the 20‐item trait anxiety scale from the State‐Trait Anxiety Inventory (STAI) (Spielberger, Gorsuch, & Lushene, 1970), which had been previously adapted to Portuguese and validated in Brazil (Biaggio & Natalício, 1979; Biaggio, Natalício, & Spielberger, 1977). The recommended cut‐off point of 40 out of 80 points was used to indicate positive screening for anxiety (Flaherman, Beiler, Cabana, & Paul, 2016; Giakoumaki, Vasilaki, Lili, Skouroliakou, & Liosis, 2009).

#### 2.1.4 Covariates

The following household characteristics were collected: per capita income (>½ Brazilian minimum wage/ >¼ ≤ ½ Brazilian minimum wage/up to ¼ Brazilian minimum wage, using the Brazilian minimum wage equivalent to 238 dollars in 2018), enrolment in the Brazilian conditional cash transfer programme (yes/no). Maternal characteristics included age (20 to 35 years/≥35 years), educational level (incomplete or complete college education or above/incomplete or complete high school/incomplete or complete elementary school), race (White/non‐White), marital status (married or living with a partner/single or divorced or separated or widowed), planned pregnancy (yes/no), wanted pregnancy (yes/no), primiparous (yes/no), type of delivery (vaginal delivery/C‐section). Infants characteristics included age (6 to 7 months/7 to 9 months/10 to 12 months), sex (male/female), preterm birth (yes/no), low birth weight (yes/no), hospitalization for more than 5 days (yes/no) or place in Neonatal Intensive Care Unit (NICU) after birth (yes/no), breastfeeding in the first hour of life (yes/no), bottle feeding (to feed any fluid including milk), and pacifier use on the prior day (yes/no). The use of bottle and pacifier were included as covariates because there is evidence that the use of artificial nipples may be a result of maternal anxiety regarding infant feeding difficulties (Batista, Rodrigues, Ribeiro, & Nascimento, 2019; Buccini, Pérez‐Escamilla, Paulino, Araújo, & Venancio, 2016; Victora, Behague, Barros, Olinto, & Weiderpass, 1997). In addition, both interfere negatively with oral facial development and language development (Carrascoza, Possobon, Tomita, & Moraes, 2006; Silveira, Prade, Ruedell, Haeffner, & Weinmann, 2013).

Continued breastfeeding and dietary diversity were assessed as recommended by the Brazilian Ministry of Health (Ministério da Saúde, 2015) based on World Health Organization recommendations for the evaluation of infant feeding practices (World Health Organization, 2008). We considered that the infant was currently being breastfed, if the mother answered ‘yes’ to the question ‘Was the infant breastfed yesterday?’. The infant was considered as having a diversified diet if he/she consumed the following six food groups on the day before: (1) breast milk, other milk, porridge with milk or yogurt; (2) fruits, vegetables and greens; (3) orange vegetables and fruits or dark green leafy; (4) meat or eggs; (5) beans; and (6) cereals or tubers (rice, potatoes, yams/cassava, flour or pasta). The dietary diversity indicator was dichotomized (yes/no) based on whether or not the child had a diversified diet the previous day.

### 2.2 Data analyses

Pearson's *χ* ^2^ test was used to evaluate the bivariate association between ECD delays and HFI, maternal depression and anxiety, and key covariates (household, maternal and infant's variables, breastfeeding and dietary diversity). Variables associated with ECD delays with *p* ≤ .20 were included in the multivariate analyses using the forced entry method. Logistic regression findings were expressed as crude and adjusted odds ratios.

To test the potential effect modification of maternal depression and anxiety on the relationship between HFI and ECD delays, we included the effect modifier variables (depression and anxiety) and their interaction with HFI in three multivariable models: Model 1 included the interaction between HFI and maternal depression; Model 2 tested the interaction between HFI and maternal anxiety; and Model 3 examined the interaction between HFI with both maternal anxiety and depression combined on ECD delays. Because the interaction was significantly associated with ECD delays in Models 1 and 3, we stratified the analyses for mothers with and without depression and with and without both depression and anxiety. Because no variable had more than 10% missing values, no data imputation was conducted. The statistical software Statistical Package for the Social Sciences (SPSS) version 20.0 was used. The level of significance used for the analyses was 5%, which is equivalent to the 95% CI.

### 2.3 Ethical considerations

This study was approved by the Research Ethics Committee of the School of Health Sciences at University of Brasília (67069417.0.0000.0030) and by the Research Ethics Committee of the Foundation of Education and Research in Health Sciences (67069417.0.3001.5553).

## 3 RESULTS

We investigated 468 mother–infant pairs. Almost half of the households were food insecure, with 8.8% of them experiencing moderate or severe HFI. Almost half (48.3%) of the mothers screened positive for anxiety and 32.3% for depression (Table 1). Most infants were aged between 7 and 9 months (46.6%) and were male (53.6%), 70.1% were breastfed in the first hour of life and 81.6% were currently breastfed. Most mothers are less than 35 years (77.1%), had not attained college education (63.5%) and were married or living with a partner (73.3%). The majority of the pregnancies (60.7%) were not planned but wanted (84.6%). The majority of births were C‐sections (55.1%), and 9.2% of the infants had low birth weight.

**TABLE 1:** Descriptive analyses of household food insecurity, maternal anxiety and depression, infant feeding and household, maternal and infant's characteristics and bivariate association of these variables with early child development delays. 2018

Study variables	All sample (%)	Early child development delays^a^		*p*
		Negative (%)	Positive *n* (%)	
Household food insecurity^b^				0.001^*^
Secure (0)	51.7	56.6	40.4	
Mild food insecurity (1–5)	39.5	37.3	44.7	
Moderate or severe food insecurity (6–14)	8.8	6.1	14.9	
Anxiety (positive >40)	48.3	42.8	61.0	<0.001^*^
Depression (positive 14–63)	32.3	29.7	38.3	0.07
Dietary diversity (yes)^c,^, ^d^	41.3	40.2	43.6	0.50
Breastfeeding in the first hour (yes)	70.1	72.2	65.2	0.13
Continued breastfeeding (yes)	81.6	82.3	80.1	0.59
Use of pacifier (yes)	33.8	32.4	36.9	0.35
Use of bottle (yes)	59.8	57.5	65.2	0.12
Monthly per capita income^d^				0.001^*^
>½ minimum Brazilian wage	59.5	65.0	46.6	
>¼ and ≤ ½ minimum Brazilian wage	24.8	22.2	30.8	
Up to ¼ of the minimum Brazilian wage	15.7	12.8	22.6	
Enrolment in the Brazilian conditional cash transfer programme (yes)	21.2	17.1	30.5	0.001^*^
Maternal age (20–35 years)	77.1	75.8	80.1	0.31
Maternal education level				<0.001^*^
Incomplete/complete college education or above	36.5	42.5	22.7	
Incomplete/complete high school	49.6	47.7	53.9	
Incomplete/complete elementary school	13.9	9.8	23.4	
Maternal race (White)	23.9	26.6	17.7	0.04^*^
Marital status (married/living with a partner)	73.3	75.2	68.8	0.15
Planned pregnancy (yes)	39.3	39.1	39.7	0.91
Wanted pregnancy (yes)	84.6	87.2	78.7	0.02^*^
First pregnancy (yes)	40.4	41.3	38.3	0.55
Type of delivery (C‐section delivery)	55.1	55.7	53.9	0.73
Infant's age				0.07
6 to 7 months	29.3	32.4	22.0	
7 to 9 months	46.6	44.4	51.8	
10 to 12 months	24.1	23.2	26.2	
Infant's sex (male)	53.6	54.7	51.1	0.46
Preterm birth (yes)^d^	8.0	6.5	11.3	0.08
Low birth weight (<2,500 kg) (yes)^d^	9.2	9.0	9.9	0.74
Infant hospitalized for more than 5 days after birth (yes)	11.8	9.5	17.0	0.02^*^
ICU after birth (yes)	6.0	5.5	7.1	0.51

Abbreviation: ICU, intensive care unit.

a Based on PIPAS's questionnaire using the 30th percentile of the score's distribution as the cut‐off point.

b Measured with the Brazilian Food Insecurity Measurement Scale (EBIA).

c Based on the indicator proposed by the Brazilian Ministry of Health. A diversified diet consists on the consumption of six food groups on the day before.

d Variables with missing values: dietary diversity (*n* = 5); monthly per capita income (*n* = 29); preterm birth (*n* = 4); low birth weight (*n* = 3).

* Statistically significant results.

In the bivariate analysis, ECD delays were associated with HFI (*p* = .001), maternal anxiety (*p* < .001), low monthly per capita income (*p* = .001), enrolment in the Brazilian conditional cash transfer programme (*p* = .001), low maternal educational level (*p* < .001), maternal non‐White race (*p* = .04), wanted pregnancy (*p* = .02) and infant hospitalization for more than 5 days after birth (*p* = .02) (Table 1).

### 3.1 Main effects models

HFI was an independent risk factor for ECD delays after controlling for covariates. Infants living in moderate or severe food insecure household had 2.52 times the odds of having ECD delays compared with infants in secure households (95% CI [1.13, 5.65]). Low maternal educational level (aOR 2.72, 95% CI [1.27, 5.85]), higher infant's age (aOR 2.01, 95% CI [1.07, 3.76]) and preterm birth (aOR 2.35, 95% CI [1.03, 5.36]) also remained significantly associated with ECD delays in the adjusted model (Table 2).

**TABLE 2:** Unadjusted and adjusted odds ratio for early child development delays, by household food insecurity and covariates. Brasília (DF). 2018

Study variables	Early child development delays^a^	Early child development delays^b,^, ^c^
	OR (95% CI)	ORaj (95% CI)^b^
Household food insecurity^d^		
Secure (0)	1	1
Mild food insecurity (1–5)	1.68 [1.10, 2.56]^e^	1.49 [0.92, 2.39]
Moderate or severe food insecurity (6–14)	3.41 [1.73, 6.73]^e^	2.52 [1.13, 5.65]^e^
Breastfeeding in the first hour		
Yes	1	1
No	1.38 [0.90, 2.11]	1.43 [0.87, 2.36]
Use of bottle		
No	1	1
Yes	1.39 [0.92, 2.09]	1.19 [0.75, 1.88]
Monthly per capita income		
>½ minimum Brazilian wage	1	1
>¼ and ≤ ½ minimum Brazilian wage	1.93 [1.20, 3.13]^e^	1.18 [0.67, 2.07]
Up to ¼ of the minimum Brazilian wage	2.47 [1.42, 4.30]^e^	1.35 [0.66, 2.75]
Enrolment in the Brazilian conditional cash transfer programme		
No	1	1
Yes	2.12 [1.34, 3.36]^e^	1.12 [0.62, 2.04]
Maternal education level		
Incomplete/complete college education or above	1	1
Incomplete/complete high school	2.11 [1.32, 3.39]^e^	1.44 [0.82, 2.54]
Incomplete/complete elementary school	4.48 [2.41, 8.32]^e^	2.72 [1.27, 5.85]^e^
Maternal race		
White	1	1
Non‐White	1.68 [1.02, 2.76]^e^	1.23 [0.70, 2.18]
Marital status		
Married/living with a partner	1	1
Single/divorced/separated/widowed	1.38 [0.89, 2.13]	1.09 [0.66, 1.80]
Wanted pregnancy		
Yes	1	1
No	1.83 [1.10, 3.08]^e^	1.53 [0.82, 2.86]
Infant's age		
6 to 7 months	1	1
7 to 9 months	1.72 [1.06, 2.81]^e^	1.70 [0.99, 2.92]
10 to 12 months	1.66 [0.95, 2.92]	2.01 [1.07, 3.76]^e^
Preterm birth		
No	1	1
Yes	1.84 [0.93, 3.64]	2.35 [1.03, 5.36]^e^
Infant hospitalized for more than 5 days after birth		
No	1	1
Yes	1.96 [1.10, 3.48]^e^	1.28 [0.63, 2.59]

Abbreviations: OR, odds ratio; CI, confidence interval.

a Odds ratio adjusted by logistic regression for monthly per capita income, enrolment in the Brazilian conditional cash transfer programme, maternal education level, maternal race, maternal marital status, wanted pregnancy, infant's age, preterm birth, infant's hospitalization for more than 5 days after birth, Household Food Insecurity, breastfeeding in the first hour of life, use of bottle.

b Based on PIPAS's questionnaire using the 30th percentile of the score's distribution as a cut‐off point.

c Hosmer‐Lemeshow statistic with *p* > .05

d Measured with the Brazilian Food Insecurity Measurement Scale (EBIA).

e Statistically significant results.

### 3.2 Interaction models

**FIGURE 1:**
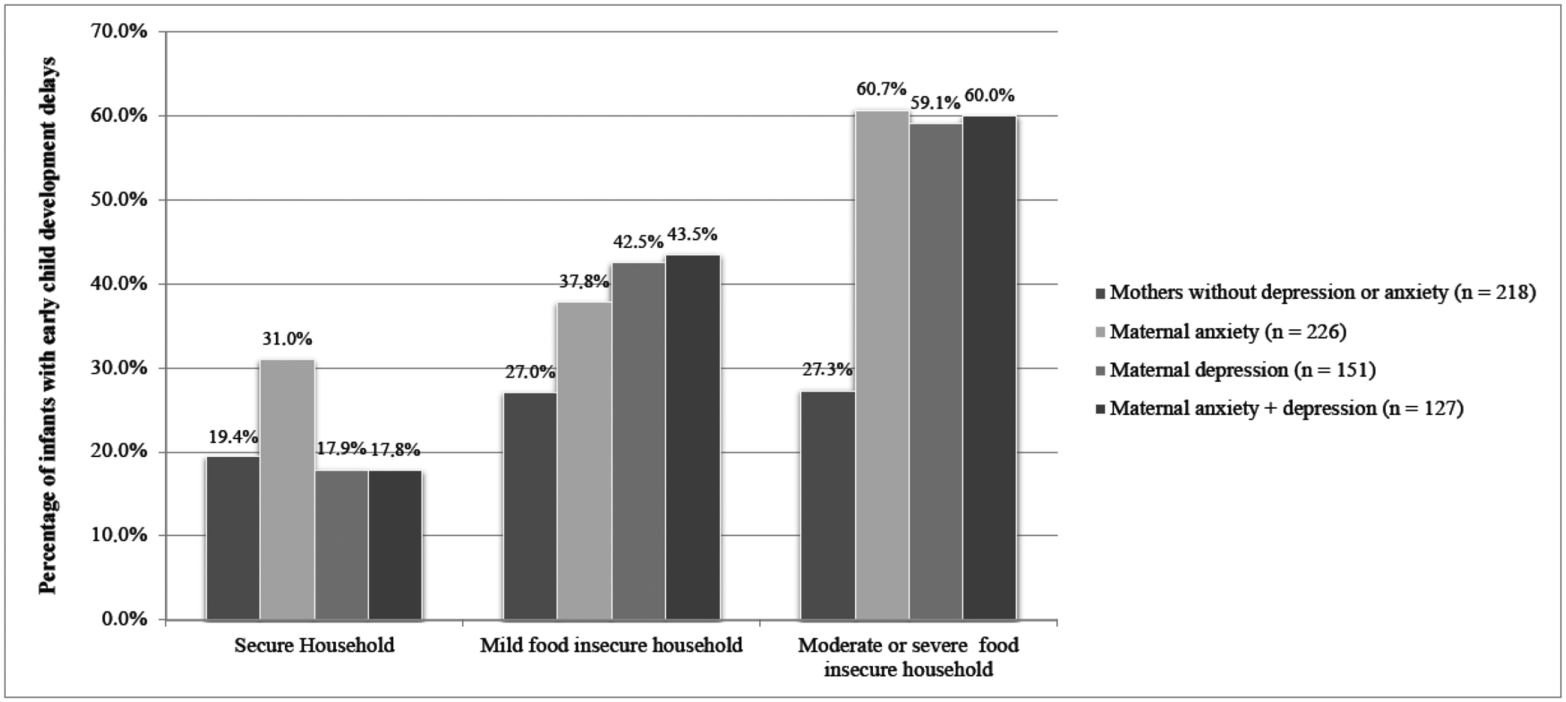
Prevalence of early child development delays, according to household food insecurity level by maternal depression, anxiety and anxiety + depression. Brasília (DF). 2018

A relationship between HFI level of severity (i.e., secure, mild insecure and moderate/severe insecure) and prevalence of ECD delays was observed among women with anxiety (*n* = 226), depression (*n* = 151) or depression and anxiety (*n* = 127) (Figure 1). The prevalence of ECD delays in moderate or severe food insecure households was twice as high in households where mothers were anxious (60.7%), depressed (59.1%) or anxious and depressed (60.0%), compared with households where the mother did not have depression or anxiety (27.3%).

Multivariable analyses suggested that maternal depression and anxiety may modify the relationship between HFI and ECD. Specifically, we found a significant interaction between HFI and maternal depression (aOR 5.85, 95% CI [1.10, 31.07]) and between HFI and maternal anxiety and depression combined (aOR 7.28, 95% CI [1.27, 41.71]) with ECD delays (Table 3). The interaction between HFI and maternal anxiety with ECD was not significant (Table 3).

**TABLE 3:** Adjusted odds ratio for early child development delays including the effect modifier variables (maternal depression, anxiety and depression + anxiety) and their interaction by household food insecurity. Brasília (DF). 2018

Study variables	Early child development delays (Model 1)^a,^, ^b^	Early child development delays (Model 2)^a,^, ^b^	Early child development delays (model 3)^a,^, ^b^
	ORaj (95% CI)^c^	ORaj (95% CI)^c^	ORaj (95% CI)^c^
Household food insecurity^d^			
Secure (0)	1	1	1
Mild food insecurity (1–5)	1.13 [0.63, 2.02]	1.57 [0.78, 3.20]	1.14 [0.65, 2.00]
Moderate or severe food insecurity (6–14)	1.26 [0.43, 3.71]	1.23 [0.32, 4.66]	1.32 [0.48, 3.66]
Depression		‐	‐
Negative	1		
Positive	0.57 [0.24, 1.33]		
Anxiety	‐		‐
Negative		1	
Positive		1.51 [0.78, 2.95]	
Depression and anxiety	‐	‐	
Negative			1
Positive			0.50 [0.20, 1.29]
Food insecurity * maternal depression		‐	‐
Security with and without depression	1		
Mild food insecurity with depression	2.57 [0.87, 7.61]		
Moderate or severe food insecurity with depression	5.85 [1.10, 31.07]^e^		
Food insecurity * maternal anxiety	‐		‐
Security with and without anxiety		1	
Mild food insecurity with anxiety		0.80 [0.31, 2.09]	
Moderate or severe food insecurity with anxiety		2.76 [0.52, 14.53]	
Food insecurity * maternal anxiety + depression	‐	‐	
Security with and without anxiety and depression			1
Mild food insecurity with anxiety and depression			3.05 [0.95, 9.81]
Moderate or severe food insecurity with anxiety and depression			7.28 [1.27, 41.71]^e^

*Note*. Model 1 included maternal depression and Food Insecurity * maternal depression as covariates. Model 2 included maternal anxiety and Food Insecurity * maternal anxiety as covariates. Model 3 included maternal anxiety + depression and Food Insecurity * maternal anxiety + depression as covariates.

Abbreviations: OR, odds ratio; CI, confidence interval.

a Based on PIPAS's questionnaire using the 30^th^ percentile of the score's distribution as a cut‐off point.

b Hosmer–Lemeshow statistic with *p* > .05.

c Odds ratio adjusted by logistic regression for monthly per capita income, enrolment in the Brazilian conditional cash transfer programme, maternal education level, maternal race, maternal marital status, wanted pregnancy, infant's age, preterm birth, infant's hospitalization for more than 5 days after birth, Household Food Insecurity, breastfeeding in the first hour of life, use of bottle.

d Measured with the Brazilian Food Insecurity Measurement Scale (EBIA).

e Statistically significant results.

Adjusted models testing effect modification of depression or both anxiety and depression revealed even stronger associations between HFI and ECD. Among infants with depressed mothers, those experiencing mild HFI had higher odds of having ECD delays (aOR 3.33, 95% CI [1.17, 9.46]) compared with infants in food secure households. In households with moderate or severe HFI, infants had 10 times the odds of having developmental delays (aOR 10.13, 95% CI [2.18, 47.10]) when the mother was depressed. Moreover, among infants whose mothers had both anxiety and depression, those experiencing mild HFI had higher odds of having ECD delays (aOR, 4.69 95% CI [1.41, 15.59]) as well as those with moderate or severe HFI (aOR 16.07, 95% CI [2.70, 95.66]), compared with those in food secure households (Table 4).

**TABLE 4:** Adjusted odds ratio for early child development delays, by household food insecurity, stratified for mothers with depression and both maternal depression and anxiety. Brasília (DF). 2018

Study variables	Early child development delays^a,^, ^b^		Early child development delays^a,^, ^b^	
	Without depression (*n* = 320)	With depression (*n* = 154)	Without depression + anxiety (*n* = 344)	With depression + anxiety (*n* = 130)
	ORaj (95% CI)^c^	ORaj (95% CI)^c^	ORaj (95% CI)^c^	ORaj (95% CI)^c^
Household food insecurity^d^				
Secure (0)	1	1	1	1
Mild food insecurity (1–5)	1.06 [0.58, 1.91]	3.33 [1.17, 9.46]^e^	1.11 [0.63, 1.96]	4.69 [1.41, 15.59]^e^
Moderate or severe food insecurity (6–14)	1.27 [0.41, 3.92]	10.13 [2.18, 47.10]^e^	1.45 [0.51, 4.15]	16.07 [2.70, 95.66]^e^

Abbreviations: OR, odds ratio; CI, confidence interval.

a Based on PIPA's questionnaire using the 30th percentile of the score's distribution as a cut‐off point.

b Hosmer‐Lemeshow statistic with *p* > .05.

c Odds ratio adjusted by logistic regression for monthly per capita income, enrolment in the Brazilian conditional cash transfer programme, maternal education level, maternal race, maternal marital status, wanted pregnancy, infant's age, preterm birth, infant's hospitalization for more than 5 days after birth, household food insecurity, breastfeeding in the first hour of life, and use of bottle.

d Measured with the Brazilian Food Insecurity Measurement Scale (EBIA).

e Statistically significant results.

## 4 DISCUSSION

The association of HFI and ECD delays has been observed in previous studies (Oliveira et al., 2020; Pérez‐Escamilla & Vianna, 2012; Shankar, Chung, & Frank, 2017). Nevertheless, a recent comprehensive systematic review pointed out that only four studies were conducted in low‐ or middle‐income countries (Oliveira et al., 2020). Oliveira et al. also verified with meta‐analysis that, in studies from high‐income countries, HFI was associated with developmental risk and poor math skills. In addition, HFI was associated with poor vocabulary skills, regardless of the income of the country where the study was conducted (Oliveira et al., 2020).

The association between HFI and ECD delays may be the result of caregivers' worries about how to provide food for the family or even the experience of hunger, resulting in fewer responsive parenting behaviours, less attachment and poor stimulation of their children's regarding cognitive and social abilities (Johnson & Markowitz, 2018a; King, 2018; Zaslow et al., 2009). In addition, in food insecure households, children are less likely to have educational books, games and toys that support development (Johnson & Markowitz, 2018a). It is important to note that in addition to HFI‐related family psychosocial dysfunctions, including caregivers' poor mental health and well‐being, HFI can also impact child's development, through micronutrient deficiencies and illnesses (Althoff, Ametti, & Bertmann, 2016; Council on Community Pediatrics & Committee On Nutrition, 2015; Greder, Peng, Doudna, & Sarver, 2017; Kimbro & Denney, 2015; King, 2018; Leung, Epel, Willett, Rimm, & Laraia, 2015; Nagata, Gomberg, Hagan, Heyman, & Wojcicki, 2018; Pérez‐Escamilla & Vianna, 2012).

We also found that maternal depression and anxiety modified the association between HFI and ECD delays. Similar findings were observed in three previous studies, but all of them were conducted in the United States (Black et al., 2012; Nagata et al., 2018; Zaslow et al., 2009). One study found that HFI in infancy was indirectly associated with cognitive and social and emotional development in toddlerhood through maternal depression and parenting practices (Zaslow et al., 2009). Another study found that children exposed to HFI and caregiver's depression had greater chance of having developmental risks (Black et al., 2012). A third study confirmed these findings (Nagata et al., 2018).

The association between HFI and maternal depression may be bidirectional (Gebreyesus et al., 2018; Weigel et al., 2016). Mothers with mental disorders may be less likely to be permanently employed or to use resources efficiently and also may spend more money addressing health problems, resulting in HFI (Althoff et al., 2016; Garg, Toy, Tripodis, Cook, & Cordella, 2015; Gebreyesus et al., 2018; Greder et al., 2017; Pérez‐Escamilla & Vianna, 2012; Weigel et al., 2016). On the other hand, HFI is a stressor, and the mother's concerns about providing food for themselves and their offspring can lead to anxiety and depression (Gebreyesus et al., 2018; Wu, Harwoodc, & Fenga, 2018). Furthermore, some micronutrient deficiencies resulting from HFI, such as folate, magnesium and zinc, may also increase the risk of maternal depression (Gebreyesus et al., 2018; Jacka, Maes, Pasco, Williams, & Berk, 2012; King, 2018). Mothers with emotional distress usually have less positive parenting skills. As a result, their caregiving behaviours can be less nurturing and responsive to the needs of the child. This lack of optimal stimulation, nurturing interactions and attachment can lead to child developmental delays (Britto et al., 2017; Johnson & Markowitz, 2018a; Johnson & Markowitz, 2018b; Milner et al., 2017; Pérez‐Escamilla & Vianna, 2012; Zaslow et al., 2009).

Therefore, given our results, we highlight the importance of HFI and ECD assessment and monitoring, both in routine health services and in national surveys (Black et al., 2017; Lu et al., 2016; Richter et al., 2017). Children living in food insecure households must be considered as being at risk for ECD delays as HFI is highly disruptive for family life. Being exposed to HFI in early childhood is a great concern because of its potential long‐term negative impact on psycho‐emotional, social and cognitive development (Black et al., 2012; Black et al., 2017; Johnson & Markowitz, 2018a). Timely screening for caregiver's anxiety and depression and HFI, and referral for needed services, must be done routinely in health and social services (Council on Community Paediatrics, 2015; Garg et al., 2015; Greder et al., 2017; Johnson & Markowitz, 2018b; Nagata et al., 2018; Shankar et al., 2017). In addition, it is important to integrate maternal–child mental health, nutrition and primary health care services through well‐designed policies and corresponding programmes (Pérez‐Escamilla & Vianna, 2012).

When ECD delays or associated risk factors, such as HFI or maternal health problems are identified, it is important to provide the child with psychosocial stimulation and access to healthy and nutritious foods to help them achieve their developmental potential and thrive (Black et al., 2017; Lu et al., 2016; Pérez‐Escamilla & Vianna, 2012). The analysis of the present study in particular points out to the importance of enforcing public policies or programmes targeting ECD that address HFI and maternal mental health in low‐ and middle‐income countries following a syndemic approach (Garcia et al., 2013). Specifically, we highlight the relevance of multisectoral intervention packages addressing nurturing care including actions/programmes to promote adequate early stimulation and responsive parenting, maternal anxiety and depression, and household food security, with special attention to the nutritional needs of young children so that they can properly learn and develop (Black et al., 2017; Britto et al., 2017; Richter et al., 2017; Sigla et al., 2015). For instance, in Brazil, the concern with ECD has increased over the past years and has gained strength with the implementation of the programme Criança Feliz. This is a home visit programme that aims to enhance parenting skills among vulnerable families, targeting pregnant women and children from birth to 6 years of age (Girade, 2018).

Our study is limited by its cross‐sectional design, which does not allow to establish the temporal sequence of events nor to draw casual inferences (Habicht, Victora, & Vaughan, 1999). Hence, although we can hypothesize it, we could not establish through our study if HFI actually preceded maternal anxiety, depression and ECD delays. Thus, it is important that moving forward, funding agencies invest more on the design of robust prospective studies in this field. Also, the sampling may limit the generalization of results to populations with different characteristics as small subgroup sample sizes resulted when stratifying the combination of different conditions of HFI, depression and anxiety. This is why the effect modification analyses yielded estimates with wide CIs. In addition, because PIPAS was designed to assess the overall level of ECD, it was not possible to report findings by developmental domains. Also, the introduction of complementary feeding should be gradual, and younger children may consume fewer groups, which could influence dietary diversity results (Bortolini, Giugliani, Gubert, & Santos, 2019). Furthermore, we did not include adolescent mothers and did not measure caregivers' practices or infants' anthropometry, which we recommend that future studies do. We also did not explore the nutritional pathway, and because there is a lack of studies that have evaluated the impact of the suboptimal nutrient intake resulting from HFI on ECD delays, future studies should address this gap (Figure S1). Despite the limitations, this research fills a knowledge gap in the literature and has many strengths, including robust effect modification analyses and the use of robust measuring tools previously validated for the Brazilian population to evaluate HFI, maternal depression, anxiety and ECD.

## 5 CONCLUSION

We found that HFI was an independent risk factor for ECD delays and that the relationship appeared to be stronger when maternal depression and anxiety were both present. Therefore, we suggest that (1) pediatricians, primary health care workers and ECD programme workers routinely screen for caregivers depression, anxiety and HFI; (2) governments invest in implementing at‐scale existing evidence‐based multisectoral intervention packages to promote caregiver's mental health and household food security as part of the ECD national agenda; (3) governments should prioritize these investments for the most vulnerable populations. However, we recognize that although our study posits plausible hypotheses, these will need to be confirmed through future prospective research and programme evaluations. This is needed to strengthen the knowledge base needed to improve the effectiveness of policies targeting HFI and maternal health following a syndemic approach (Garcia et al., 2013). Therefore, longitudinal cohort studies are urgently needed to further understand the relationship between HFI and ECD delays; how it gets modified by maternal health problems in low‐ and middle‐income countries; and whether interventions that promote caregiver's mental health and HFI impact ECD.

## CONFLICTS OF INTEREST

The authors have no conflicts of interest relevant to this article to disclose.

## CONTRIBUTIONS

JP and MBG designed the research study. RPE contributed substantially to the development of the conceptual and analytical framework. JP performed the research. SIV designed the child development data collection instrument. JP, GB, SIV, RPE and MBG analysed and interpreted data. JP drafted the initial manuscript. GB, SIV, RPE and MBG critically reviewed the manuscript for important intellectual content. All authors have read and approved the final manuscript.

## Supporting information


**Figure S1:** Supporting Information

**Table S1.** Percentage of positive items for early child development measured by the Early Childhood for Healthy Adults Questionnaire's (PIPAS) by household food insecurity, maternal depression and anxiety. Brasília (DF). 2018.
