# Supplementary material for: Maternal mental health modifies the association of food insecurity and early child development
Source: Matern Child Nutr. 2020 Apr 30;16(4):e12997. doi: 10.1111/mcn.12997 (PMC7507582; doi:10.1111/mcn.12997)
Supplement: Supplementary file 1 — Figure S1: Supporting Information [file MCN-16-e12997-s001.pdf]

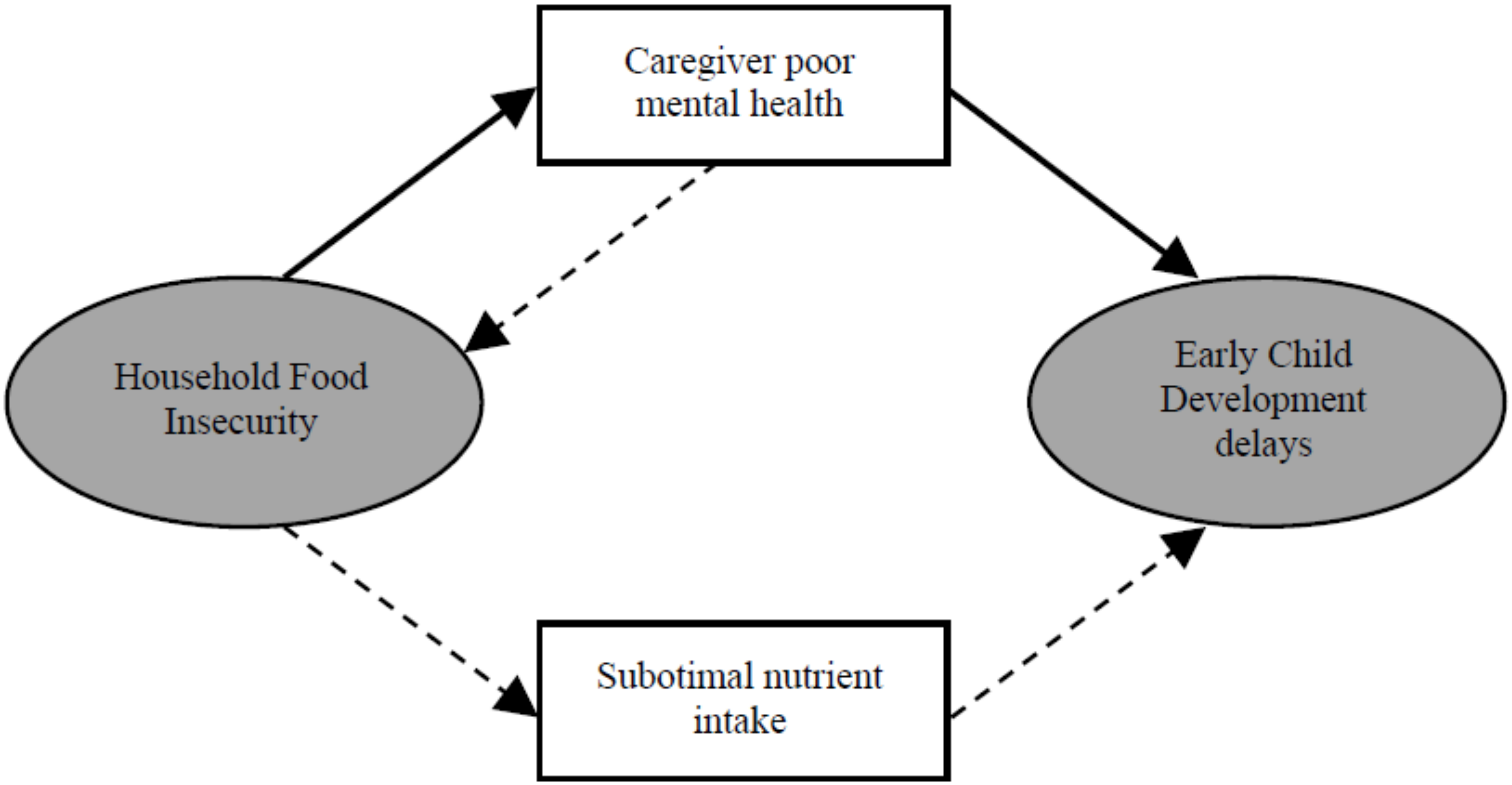

Adapted from Pérez-Escamilla & Vianna (2012).
Solid lines indicate the pathways analyzed in the present study, and dashed lines indicate pathways that were not analyzed.
