# Supplementary material for: Maternal mental health modifies the association of food insecurity and early child development
Source: Matern Child Nutr. 2020 Apr 30;16(4):e12997. doi: 10.1111/mcn.12997 (PMC7507582; doi:10.1111/mcn.12997)
Supplement: Supplementary file 2 — Table S1. Percentage of positive items for early child development measured by the Early Childhood for Healthy Adults Questionnaire's (PIPAS) by household food insecurity, maternal depression and anxiety. Brasília (DF). 2018. [file MCN-16-e12997-s002.docx]

| Supplementary Table 1. Percentage of positive items for early child development measured by the Early Childhood for Healthy Adults Questionnaire’s (PIPAS) by household food insecurity, maternal depression and anxiety. Brasília (DF). 2018. | | | | | | | | |
| --- | --- | --- | --- | --- | --- | --- | --- | --- |
|  | All sample | Household Food Insecurity | | | Maternal depression | | Maternal anxiety | |
|  |  | **Secure** | **Mild Food Insecure** | **Moderate or severe Food Insecure** | **No** | **Yes** | **No** | **Yes** |
|  | **%** | **%** | **%** | **%** | **%** | **%** | **%** | **%** |
| ITEMS |  |  |  |  |  |  |  |  |
| 6 to 6 months and 30 days |  |  |  |  |  |  |  |  |
| Does the infant, when lying on his/her back, move his/her arms and legs? | 100.0 | 100.0 | 100.0 | 100.0 | 100.0 | 100.0 | 100.0 | 100.0 |
| When lying on the tummy, can the infant keep his/her head up? | 100.0 | 100.0 | 100.0 | 100.0 | 100.0 | 100.0 | 100.0 | 100.0 |
| Can the infant put his/her hands together and hold a toy? | 100.0 | 100.0 | 100.0 | 100.0 | 100.0 | 100.0 | 100.0 | 100.0 |
| Does the infant respond to sounds? | 99.3 | 98.7 | 100.0 | 100.0 | 100.0 | 97.8 | 100.0 | 98.2 |
| When you smile and talk to the infant, does he/she respond with smile and sounds? | 100.0 | 100.0 | 100.0 | 100.0 | 100.0 | 100.0 | 100.0 | 100.0 |
| Does the infant try to talk to you? | 100.0 | 100.0 | 100.0 | 100.0 | 100.0 | 100.0 | 100.0 | 100.0 |
| Does the infant fix and follow your face with his/her eyes? | 99.3 | 100.0 | 98.1 | 100.0 | 98.9 | 100.0 | 98.8 | 100.0 |
| Does the infant search for your look? | 100.0 | 100.0 | 100.0 | 100.0 | 100.0 | 100.0 | 100.0 | 100.0 |
| Is it hard to calm the infant when he/she's crying, even when you pick him/her up? † | 77.0 | 88.5 | 63.0 | 57.1 | 78.5 | 73.9 | 80.5 | 71.9 |
| 7 months to 9 months and 30 days |  |  |  |  |  |  |  |  |
| When lying down, can the infant completely turn around? | 98.2 | 100.0 | 97.7 | 91.3 | 99.3 | 95.7 | 100.0 | 96.2 |
| Does the infant pass a toy or object from one hand to the other? | 100.0 | 100.0 | 100.0 | 100.0 | 100.0 | 100.0 | 100.0 | 100.0 |
| Is the infant able to sit without the support of the hands to balance? | 96.8 | 98.1 | 95.3 | 95.7 | 97.3 | 95.7 | 98.2 | 95.2 |
| Does the infant imitate the sounds you make when you talk to her? | 92.6 | 90.7 | 94.2 | 95.7 | 91.9 | 94.2 | 92.0 | 93.3 |
| Does the infant locate sounds? | 100.0 | 100.0 | 100.0 | 100.0 | 100.0 | 100.0 | 100.0 | 100.0 |
| When an object falls to the ground, does the infant look with its eyes? | 99.5 | 99.1 | 100.0 | 100.0 | 99.3 | 100.0 | 99.1 | 100.0 |
| Is it hard to calm the infant when he/she's crying, even when you pick him/her up? † | 78.8 | 85.2 | 74.4 | 65.2 | 80.4 | 75.4 | 87.5 | 69.5 |
| Does the infant show when they like something or not? | 98.2 | 99.1 | 97.7 | 95.7 | 98.6 | 97.1 | 99.1 | 97.1 |
| Does the infant accept food that is pasty or in pieces? | 99.1 | 99.1 | 100.0 | 95.7 | 99.3 | 98.6 | 100.0 | 98.1 |
| 10 to 12 months |  |  |  |  |  |  |  |  |
| Can the infant get up by leaning / holding somewhere? | 97.3 | 96.4 | 100.0 | 90.9 | 98.7 | 94.4 | 100.0 | 95.3 |
| Can the infant get small objects with his/her thumb and index finger? | 98.2 | 98.2 | 100.0 | 90.9 | 98.7 | 97.2 | 100.0 | 96.9 |
| Can the infant take a few steps with support? | 92.9 | 92.9 | 97.8 | 72.7 | 93.4 | 91.7 | 93.8 | 92.2 |
| Does the infant call "mama" or "dada" or similar name? | 95.5 | 96.4 | 95.6 | 90.9 | 97.4 | 91.7 | 95.8 | 95.3 |
| Does the infant understand requests such as "Come here" or "Give me the toy"? | 97.3 | 98.2 | 95.6 | 100.0 | 98.7 | 94.4 | 95.8 | 98.4 |
| Does the infant look at you when you call him/her by name? | 100.0 | 100.0 | 100.0 | 100.0 | 100.0 | 100.0 | 100.0 | 100.0 |
| Does the infant play peek-a-boo? | 90.2 | 91.1 | 91.1 | 81.8 | 92.1 | 86.1 | 91.7 | 89.1 |
| Does the infant imitate when you clap or wave? | 99.1 | 98.2 | 100.0 | 100.0 | 100.0 | 97.2 | 100.0 | 98.4 |
| Does the infant strange people unknown to him/her? | 67.0 | 62.5 | 68.9 | 81.8 | 64.5 | 72.2 | 62.5 | 70.3 |
| Is it hard to calm the infant when he/she's crying, even when you pick him/her up? † | 72.3 | 80.4 | 68.9 | 45.5 | 76.3 | 63.9 | 79.2 | 67.2 |
| Does the infant show when they like something or not? | 98.2 | 98.2 | 97.8 | 100.0 | 98.7 | 97.2 | 100.0 | 96.9 |
| Does the infant ask to be held for familiar people? | 74.1 | 67.9 | 86.7 | 54.5 | 75.0 | 72.2 | 77.1 | 71.9 |
| All ages |  |  |  |  |  |  |  |  |
| Is it hard to calm the infant when he/she's crying, even when you pick him/her up? † | 76.7 | 85.1 | 69.7 | 58.5 | 78.9 | 72.2 | 83.5 | 69.5 |

† Items in the negative early child development direction.
